# Effect of NaCl road salt on the ionic composition of soils and *Aesculus hippocastanum* L. foliage and leaf damage intensity

**DOI:** 10.1038/s41598-021-84541-x

**Published:** 2021-03-05

**Authors:** Katarzyna Łuczak, Izabela Czerniawska-Kusza, Czesława Rosik-Dulewska, Grzegorz Kusza

**Affiliations:** 1 Department of Land Protection, University of Opole, Oleska 22, 45-052 Opole, Poland; 2 Institute of Biology, University of Opole, Oleska 22, 45-052 Opole, Poland; 3 Institute of Environmental Engineering Polish Academy of Sciences, M. Skłodowska-Curie 34, 41-819 Zabrze, Poland

**Keywords:** Plant sciences, Environmental sciences

## Abstract

We investigated the accumulation of sodium chloride in roadside soils and common horse chestnut *Aesculus hippocastanum* L. under urban conditions to evaluate changes in soil and leaf ionic content and their relationship with foliar damage, considering the visual assessment of trees of the same health status. A total of 15 field sites were assessed in late June 2016. The analysis included soil granulometric composition, pH, electrical conductivity, and the content of Cl^−^, Na^+^, K^+^, Ca^2+^, and Mg^2+^ ions in soil and foliage samples. The results showed increased salinity and alkalization of roadside soils together with the decreased magnesium content. Foliage samples manifested significantly higher concentrations of Na^+^ and Cl^−^. A wide range of Cl^−^ content was noted in leaves (2.0–11.8% d.w.) regardless of their damage index. On the contrary, leaf damage was strongly correlated with increasing Na^+^ concentrations and decreasing K^+^ and Mg^2+^. A severe imbalance of nutrients, and therefore poor urban tree vitality, can be attributed to the excessive accumulation of de-icing salt. However, further research would be needed to clarify the discrepancy between the extent of leaf damage and chloride content.

## Introduction

In cold-climate regions, winter road safety and mobility are of primary concern. Hence, with progressive urban development and road network expansion, the amount of de-icers used for road safety maintenance has increased proportionately. A variety of chemical substances have been used for snow- and ice-melting operations, from chlorides to organic compounds^1^. Among them, chloride salts have been used most widely for decades, particularly NaCl, because of its abundance, effectiveness and low cost^2,3^. De-icing salts enter the adjacent roadside environment through different pathways such as runoff, infiltration and airborne spreading and then become available to plant roots or the underlying water table^4,5^.

Prolonged use of de-icing salts has a detrimental impact on the environment, most of all on roadside soils and vegetation^6–8^. Abundant evidence demonstrates that elevated concentrations of NaCl ions in the soil solution both directly and indirectly alter plant growth and affect the health status of roadside trees^9,10^. Though plants perceive and respond to salt-induced stress by quickly altering gene expressions in parallel with biochemical and physiological changes^11^, prolonged exposure to salts weakens their defence mechanisms. Consequently, Na^+^ and Cl^−^ excessively accumulated in leaf tissue cause direct toxicity through disturbances of metabolic processes and ionic steady state at the cellular level^12–14^. Moreover, plant injury can be intensified by salt-induced water stress, producing disruption in normal water and nutrient uptake^15^. The intensity of the disruption in the basic nutrient uptake and plant growth is also species-dependent^16^. The typical injury symptoms appear as leaf discolouration (yellowing and browning) beginning at the margin and progressing towards the midrib along with increasing salt content, which leads to premature senescence and foliar loss. As a general rule, injury symptoms tend to occur once leaf chloride content exceeds 1% of dry weight in deciduous tree species although variations exist in the literature^17,18^. De-icing salts have been shown to also indirectly affect plant growth via perturbations of soil properties and plant nutrient or heavy metal bioavailability. High Na^+^ concentrations tend to displace naturally occurring cations, including important plant nutrients such as Zn, Cu, K and Mn as well as disperse soil colloids, reducing soil permeability and aeration and increasing surface runoff and erosion rate^5,19^. Meanwhile, high Cl^−^ concentrations increase the mobility of heavy metals in the soil matrix^20,21^.

Several field and experimental studies have been conducted on the effects of NaCl on trees, focusing on various issues such as plant tolerance and biochemical and morphological changes, including seedlings and mature trees as well as environmental factors affecting the intensity of salt impact^3,4,22–24^. Although trees growing in an urban environment are exposed to multiple stresses because of unfavourable microclimate conditions, air pollution, poor surface permeability or mechanical injuries^25^, long-term monitoring studies in the city of Edmonton (Canada) confirmed that both soil salinity and salt spray deposition are among the main abiotic factors contributing to the decline of urban trees^26^. Generally, an excessive accumulation of de-icing salt attributes to poor physiological traits and impoverishment of mycorrhizal symbiosis^27^, which suppress the growth rate and vitality of urban trees.

This research examined the effects of NaCl de-icing salt on roadside soils and common horse chestnut *Aesculus hippocastanum* L. The objectives of our study were to determine (1) what changes occurred in the analysed soil properties, (2) whether foliar damage was associated with the use of sodium chloride, (3) to what extent the variation in severity of leaf injury observed within the single trees indicates a similar variation in leaf ionic accumulation and (4) how the disruption of ionic balance affected the intensity of leaf blade damage.

## Material and methods

### Site and sampling

The study was conducted in Opole City (50°40′0″N, 17°56′59″E), southern Poland. The city covers an area of 96 km^2^ and currently has more than 130,000 inhabitants. The climate in this area is mainly continental, moderately warm and humid. The mean annual precipitation is 611 mm. The mean monthly temperature is 19.2 °C for the warmest month (July) and − 1.9 °C for the coldest one (January). The length of growing season is about 230 days^28^. Within the city area, the dominant soil type is rendzinas formed on carbonate rocks.

**Figure 1:**
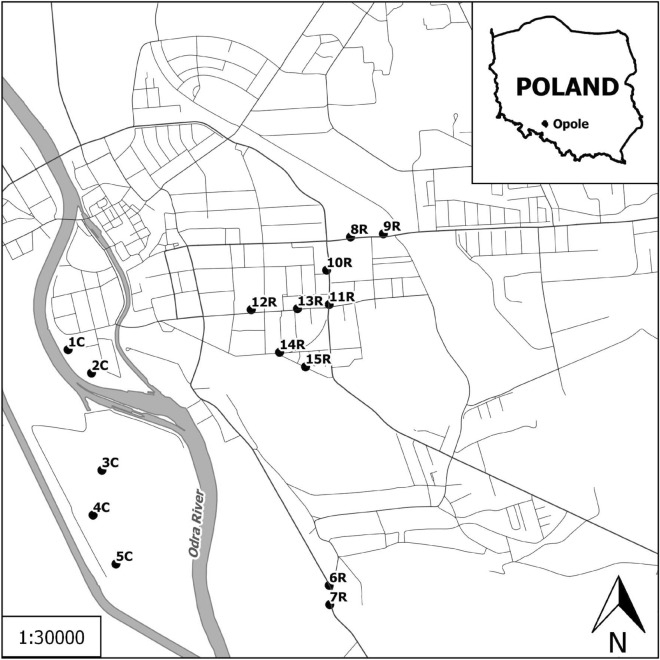
The geographic position of Opole and the location of the studied sites. *C* park, *R* roadside (The map has been made based on OpenStreetMap data, using QGIS 2.14. Essen).

A total of 15 sites with horse chestnut tree *Aesculus hippocastanum* L. were surveyed in the centre of Opole: 10 high-traffic roadside sites with salt application and 5 situated in municipal parks used as controls (Fig. 1). Street sections were comparable in terms of traffic intensity and environmental conditions (sunlight exposure conditions, roadside dimension and ground surface within the vertically projected tree crown area). Annual average daily traffic volume ranged from approximately 9000 to 10,700 vehicles (unpublished data from the Municipality of Opole). The horse chestnut trees were located 1–2 m from the road edge.

Soil and foliage samples were collected in late July 2016. Soil samples were collected from two depths (0–10 and 10–30 cm) under the tree canopy and at the same distance from the road (0.5–1.0 m) in each site. For each soil sample, 10 subsamples were obtained and thoroughly mixed. Roadside and park trees of *A. hippocastanum*, of the same age (50–55 years old) and with similar visually evaluated health status, were selected for the study since the species has been often used in urban plantings. The evaluation included the following symptoms: leaf chlorosis and necrosis, dieback of twigs and branches, and stem injury.

Foliage samples were collected from the whole tree height, if possible, and from different positions in the tree crown as well as from a well-lit portion representative of the overall crown condition of the tree. From each roadside tree, seven foliage samples containing 50 leaves each were collected and put into paper bags. Leaves were separated, directly in the field, into seven groups based on damage magnitude: index 0—healthy, 1—visible minor damage of the leaf blade up to 5%, 2—necrotic symptoms from 5 to 15%, 3—from 15 to 25%, 4—from 25 to 50%, 5—from 50 to 75%, index 6—more than 75% of the leaf blade necrotic.

### Sample treatment and analysis

Soil samples were air-dried and sieved (2.0 mm). Foliage samples were also air-dried and then pulverised with a Retsch ball mill. Soil physical and chemical properties were analysed according to methods described by Ostrowska et al.^29^. Granulometric composition was determined by modified Casagrande aerometric method. Soil pH was measured in a 1:2.5 soil/distilled water mixture and in a 1:2.5 soil/KCl 1 M mixture. Electrical conductivity was measured in a 1:5 soil/distilled water extract. Cl^−^ content was analysed using the argentometric titration method. Chloride ion concentrations in the plant material were determined by modified Piper’s method. Soil and foliar concentrations of extractable cations (Na^+^, K^+^, Ca^2+^, Mg^2+^) were analysed with flame atomic absorption (BWB-XP flame photometer) or atomic absorption spectroscopy (Thermo iCE 3500 model) after the previous extraction with ammonium acetate.

Differences in soil chemical properties (pH, EC, Cl^−^, Na^+^, K^+^, Ca^2+^, Mg^2+^) between roadside and park samples were tested using the Mann–Whitney U test. The strength of relationships between individual variables (soil nutrients vs. leaf nutrients, leaf nutrients vs. injury index) was examined using Spearman correlations (R). To determine the differences among nutrient concentrations in leaves with various injury intensity, the Kruskal–Wallis test was performed. Weighted pair-group method using the centroid average (WPGMA) cluster analysis was performed for foliage sample groups considering differences in leaf damage intensity. All statistical analyses were done using STATISTICA software (version 13.1).

## Results

### Soil characteristics

Table 1 shows the granulometric composition of the investigated soils. For all sampling plots, the sandy fraction constituted the vast majority in the soil, with minimal clay fraction. The pH indicator values, measured both in H_2_O and KCl, were higher in the roadside soil samples compared with the control ones (Table 2). Roadside soil pH was alkaline ranging from 7.8 to 8.5 in H_2_O and 7.3 to 7.4 in KCl. The same trend was evident for electrical conductivity, as EC values were twice as high as control values. In general, roadside and park soils differed significantly (p < 0.001) in both parameters. Moreover, values of soil pH (in H_2_O) strongly correlated with electrical conductivity (p < 0.001) (n = 30): R = 0.80 for samples of 0–10 cm depth and R = 0.65 for samples of 10–30 cm depth.

**Table 1:** Granulometric composition of soil samples (values are min–max).

Soil samples	Depth (cm)	Percentage content of fractions with diameter (mm)				
		> 2	< 2	2–0.05	0.05–0.002	< 0.002
Roadside (n = 10)	0–10	13–24	76–87	89–93	6–10	1
	10–30	25–44	56–79	85–91	8–11	3
Park (n = 5)	0–10	14–18	82–86	90–93	7–8	1–2
	10–30	15–38	62–85	78–80	10–18	4–10

**Table 2:** The pH indicator, conductivity (EC), chloride and cation concentrations in soils.

Soil samples	Depth (cm)	pH		EC (µS cm^−1^)	Cl^−^	Na^+^	K^+^	Mg^2+^	Ca^2+^
		H_2_O	KCl		(mg 100 g^−1^ d. m.)				
Roadside (n = 10)	0–10	7.8–8.1	7.3–7.4	171–298	1.67 (0.69)	17.9 (6.1)	3.65 (2.11)	0.44 (0.32)	3.72 (0.55)
	10–30	8.0–8.5	7.3–7.4	239–419	1.84 (0.58)	16.2 (2.6)	1.85 (0.6)	0.13 (0.18)	3.30 (0.52)
Park (n = 5)	0–10	7.5–7.7	6.9–7.3	90–130	0.39 (0.08)	2.36 (1.0)	4.52 (0.85)	1.76 (0.12)	1.67 (0.13)
	10–30	7.6–7.8	6.9–7.3	126–198	0.66 (0.14)	0.57 (0.11)	2.42 (0.17)	0.67 (0.03)	4.64 (0.22)
Statistical test (n = 15)	0–10	*	*	*	*	*	n.s	*	*
	10–30	*	n.s	*	*	*	n.s	*	*

Values are: min–max (pH, EC), median, and IQR in parentheses (Cl, Na, K, Mg, Ca).

*p < 0.001 significant difference between roadside and park samples (Mann–Whitney *U* test).

Chloride and exchangeable cation concentrations in the soil solution varied except for K^+^ ions, which were present in small amounts, further decreasing with the depth of the soil profile (Table 2). Both chloride and sodium concentrations were elevated in roadside soils, increasing slightly with soil depth. In the surface layer, the average values of Na^+^ and Cl^−^ concentrations were seven and four times higher, respectively, compared with the control soils. On the contrary, Mg^2+^ concentration revealed a fourfold decrease. Roadside soils were rich in calcium, which average concentration was twice as high as in control soils from the park, but only in the surface layer to a depth of 10 cm. However, with the depth of the soil profile, the Ca^2+^ concentration slightly decreased, in contrast to its increasing level in the control soils. The concentrations of chloride and exchangeable cations (except K^+^) showed a significant difference (p < 0.001) between roadside and park soils.

Only Cl^−^ and Ca^2+^ soil concentrations were significantly correlated with the concentrations of these ions in the leaves (R = 0.65 and R = 0.27, respectively, p < 0.001) (n = 90). Complete and detailed soil data are in the Supplementary Appendix.

### Foliar damage and chemistry

Visual inspection of horse chestnut trees, carried out from April to August, helped to identify leaf development changes. On apparently healthy branches with normally developed leaf blades, leaves began to show injury symptoms in mid-June onwards. The most common symptoms were chlorosis and necrosis of leaf margins. Foliar damage (5–15%) spread, and by August, necrosis had reached over 75% of the leaf blade, and premature leaf fall occurred. All the above visual features, from excellent health and no leaf scorch to severe leaf damage, were found in every roadside tree.

**Figure 2:**
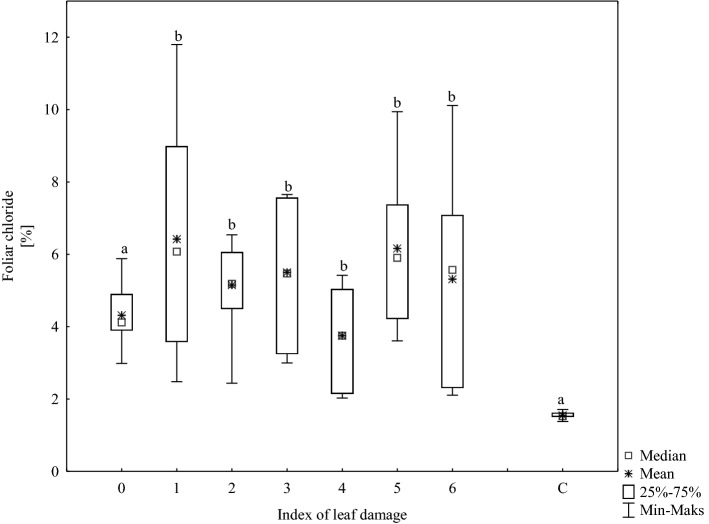
Chloride concentrations in foliage samples. C = park trees (control, n = 5), 0–6 = roadside trees (n = 10 per leaf damage severity). Results with the same letter are not statistically different (Kruskal–Wallis test, p < 0.05).

In all analysed samples, Cl^−^ and Na^+^ concentrations in the leaves of roadside trees were significantly higher than in those of park trees. The chloride ion concentration in leaf tissues ranged from 2.0 to 11.8% compared with 1.4 to 1.7% for controls (Fig. 2). Even leaves showing no outward signs of damage (index 0) had high chloride levels, as it was between 3.0 and 5.9% with an average value of 4.3%. In addition, surprisingly, there was no visible difference in Cl^−^ concentration in leaves that differed in leaf blade damage. Chloride content varied widely both in foliage samples showing a low degree of leaf blade injury (up to 5%) and those severely damaged (over 75%); consequently, Cl^−^ concentration and injury intensity was not correlated in roadside trees.

**Figure 3:**
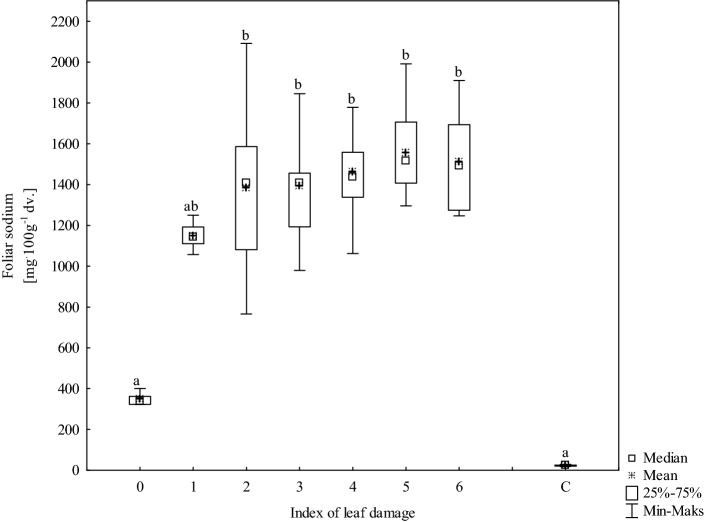
Sodium concentrations in foliage samples. C = park trees (control, n = 5), 0–6 = roadside trees (n = 10 per leaf damage severity). Results with the same letter are not statistically different (Kruskal–Wallis test, p < 0.05).

Sodium ion concentration was also significantly elevated in the leaves with no visible signs of damage. For leaves with index 0, Na^+^ concentration ranged from 323 to 401 mg 100 g^−1^ d.w. compared with 18.7 to 29.1 mg 100 g^−1^ d.w. in the control samples (Fig. 3). However, in leaves with various damage symptoms (index 1–6), Na^+^ ion concentration sharply increased, reaching values from 766 to 2092 mg 100 g^−1^ d.w., which were as much as 42–100 times higher than those of the controls. In addition, the degree of leaf blade damage increased with increasing Na^+^ concentration. A strong correlation between Na^+^ concentration and degree of leaf blade damage was observed, R = 0.67 (p < 0.001) (n = 70).

Along with the increase in Na^+^ concentration in particular leaf damage classes, a downwards trend was also noted for other cations (Table 3), especially K^+^ whose median concentration ranged from 1618 (index 0) to 873 mg 100 g^−1^ d.w. (index 5), drastically decreasing to 125 mg 100 g^−1^ d.w. in leaves with damage symptoms reaching over 75% of the leaf blade (index 6). Both K^+^ and Mg^2+^ concentrations were significantly correlated with leaf necrosis but revealed a negative relationship (R = − 0.86 and R = − 0.61, p < 0.001, respectively).

**Table 3:** Basic cation concentrations in leaves of roadside trees (mg 100 g^−1^).

Cation	Index of leaf injury							R (n = 70)
	0	1	2	3	4	5	6	
K^+^	1618 (155)	1335 (353)	1265 (171)	1001 (83)	1036 (93)	873 (104)	125 (53)	− 0.86
Mg^2+^	178 (43)	200 (43)	201 (67)	166 (19)	143 (52)	129 (4)	124 (53)	− 0.61
Ca^2+^	1331 (143)	2135 (1262)	1711 (1429)	1345 (1056)	1761 (548)	1072 (609)	1107 (709)	n.s.

Correlation between cation concentrations and leaf injury index (Spearman correlation coefficient, p < 0.001).

Values are median and IQR in parentheses (n = 10 per leaf damage severity).

**Figure 4:**
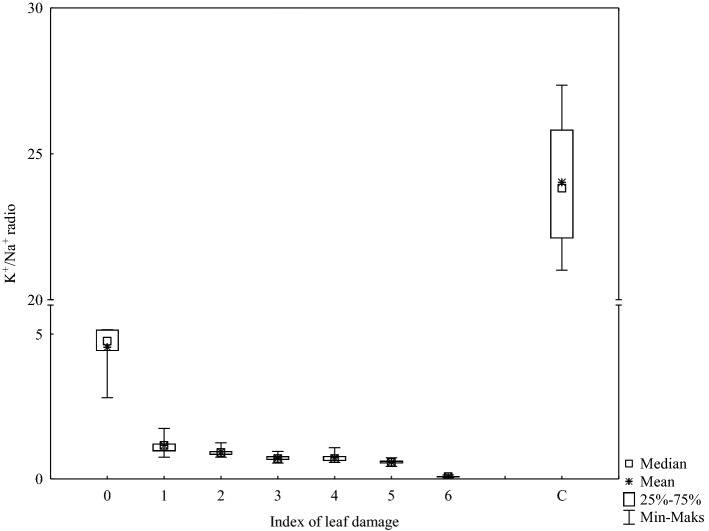
K^+^/Na^+^ ratio in different foliage samples. C = park trees (control, n = 5), 0–6 = roadside trees (n = 10 per leaf damage severity).

Figure 4 illustrates differences in the ratio of Na and K cation concentration in leaves. In the leaves of park trees, the K^+^/Na^+^ ratio was high, and the obtained indicator values were in a wide range from 22 to 25.5 for most analysed samples. Meanwhile, for horse chestnut trees growing along the streets, the K^+^/Na^+^ ratio was at least four times lower in the leaves without visible damage symptoms. In addition, a further gradual decrease in indicator value was recorded, eventually reaching 0.5 in leaves with severely damaged leaf blades (> 75%). Similar results were obtained for the Ca^2+^/Na^+^ ratio though the calcium level in the foliage of roadside trees was much higher than in park trees.

**Figure 5:**
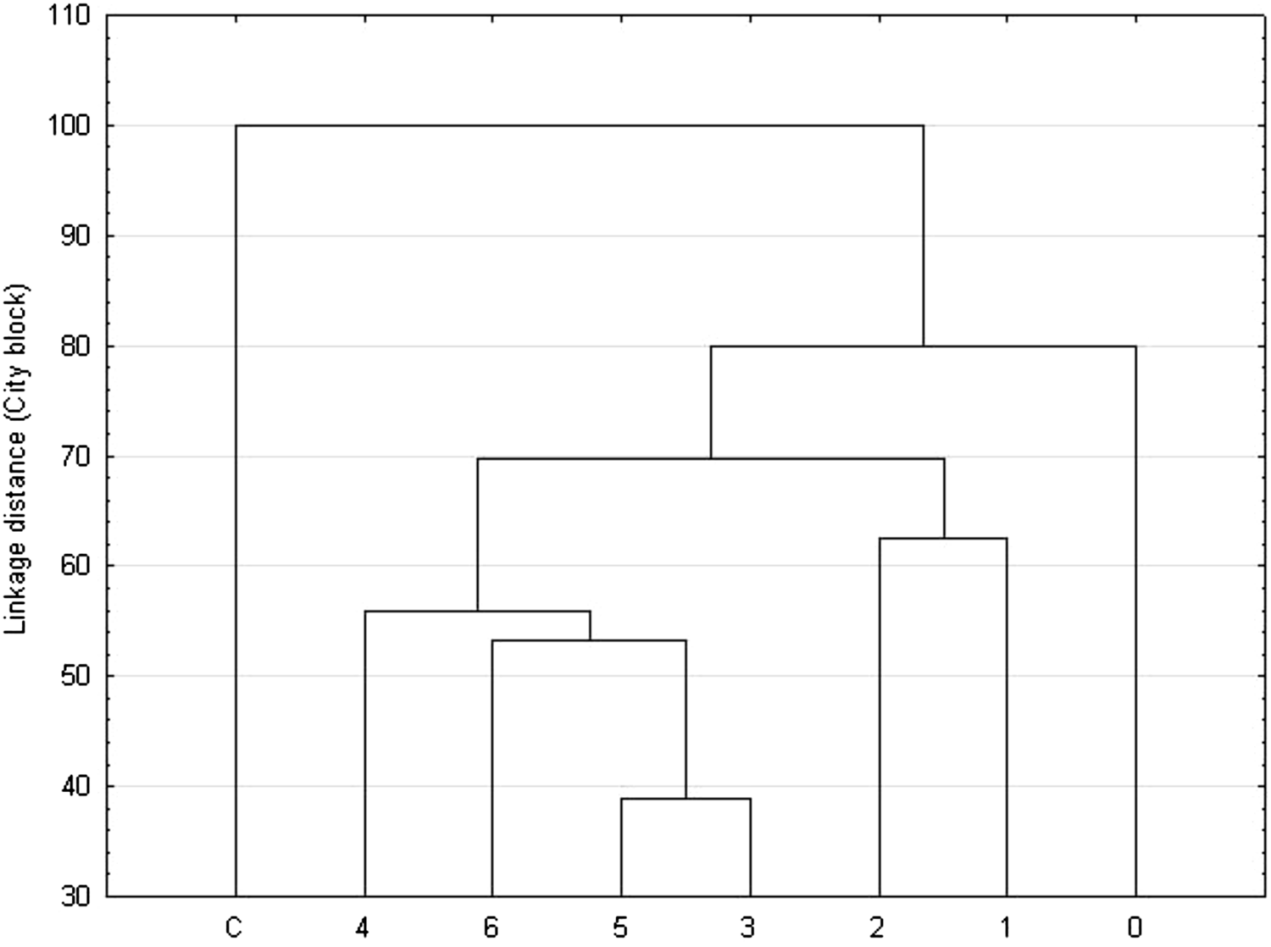
Dendrogram of cluster analysis (WPGMA) showing the similarities of foliage samples based on the chemical composition. C = park trees (control, n = 25), 0–6 = roadside trees (n = 50 per leaf damage severity).

Nutrient analysis differentiated and grouped horse chestnut leaves (Fig. 5). Based on chloride and cation concentrations, four separate groups were identified: 1—healthy (park trees), 2—no visible damage (roadside trees), 3—up to 15% leaf blade damage, 4—over 15% leaf blade damage.

## Discussion

Sodium chloride used for snow and ice control has been shown to affect roadside soils and threaten plant health and development. Many times higher Cl^−^ and Na^+^ concentrations in roadside soils compared with park soils might be attributed to de-icing salt accumulation. Among the exchangeable base cations (Ca^2+^, Mg^2+^ and K^+^), only lower magnesium content was observed in the investigated roadside soils. Elevated concentrations of chloride and sodium ions, along with a decrease in the content of other naturally occurring cations, are typical changes in soil chemical composition when exposed to de-icing salts. Several studies provide evidence of ion exchange in the soil between Na^+^ and important basic nutrients such as Ca^2+^ and Mg^2+^^7,30,31^ and K^+^^32^. The low concentration of these plant nutritive cations may result from both the increased competition with sodium for the remaining cation exchange sites and lowered concentrations of clay particles and soil organic matter, and thus from the low roadside soil's cation exchange capacity^33^. Magnesium loss not only affects soil fertility but can additionally affect its structure since Mg^2+^ cations have been found to increase soil stability, permeability and aeration, probably by flocculation of organic and inorganic particles^34^.

Another factor affecting the health conditions of roadside trees was soil pH, alkaline in all the analysed samples. Disturbed urban soils typically exhibit elevated pH because of the weathering of calcareous building materials and other waste materials as well as alkaline ash pollution, but this is highly site-specific depending on land-use history^35,36^. It should be noted that rendzinas developed on marls and limestone rocks predominate in the Opole region; therefore, their natural condition exceeds pH 6.5. However, pH values recorded for roadside soils were much higher compared with park soils, indicating further alkalization of saline urban soils. This phenomenon is affirmed in many studies evaluating the de-icing salt impact on the urban environment^23,37,38^. Marosz^39^, investigating roadside soils in central Poland, reported a significant increase in soil pH, from slightly acidic to alkaline, associated with the de-icing salt application. The highest pH values were at a distance of up to 2 m from the road edge, then gradually decreased. We collected soil samples from the nearest vicinity to the road line, thus excessive Na^+^ accumulation and elevated soil pH. High soil pH can limit the rates of key microbial N transformation processes and nutrient availability for plants^40,41^. According to Puchalski and Prusinkiewicz^42^ pH values in the soil surface layer (7.8–8.0) significantly exceeded the optimal range recommended for *A. hippocastanum* (pH 6.6–7.2). Consequently, the uptake of micro- and macronutrients important for plant physiology, such as P, Mn, Cu and Fe, can be hampered.

Sodium and chloride accumulation was clearly observed in the leaves of roadside horse chestnut, together with necrotic changes, which was associated with the application of NaCl as a street de-icer. This observation is consistent with many previous literature data^43,44^ though the concentrations obtained in Opole, on calcium-rich soils, were much higher. Conflicting reports exist regarding salt tolerance in many plant species, including *A. hippocastanum*. Dobson^2^ provided an overview of 332 woody species’ relative tolerance to salt. Horse chestnut has been described as sensitive as well as tolerant. Based on many reports, the genus *Aesculus* has been listed among deciduous trees sensitive to soil salinity and salt spray, with a threshold for salt damage of 0.3–0.5% d.w. during early summer^45^. There are many reasons for this discrepancy. Both soil type and climate variability can cause differences in plant response between areas. Tolerance may also vary depending on salt exposure method (salt spray vs. soil salt). In addition, the results derived from field observations also differ from those obtained from experimental studies and controlled NaCl application. This may also be due to differences in salt stress sensitivity between seedlings and mature trees. Overall, *A. hippocastanum* appears to have an intermediate ability to tolerate sodium and chloride from de-icing salt^2,37^.

Although chloride is an essential plant micronutrient that regulates enzyme activities in the cytoplasm, is a co-factor in photosynthesis and plays the major role in turgor pressure generation and cell expansion^46,47^ excessive amounts may cause specific ion toxicity and plant injury^48^. The critical tissue Cl^−^ concentration for toxicity is about 4–7 and 15–50 mg g^−1^ d.w. for Cl^–^-sensitive and Cl^–^-tolerant plant species, respectively^49^. *A. hippocastanum* dieback symptoms frequently occur when the Cl^−^ concentration exceeding 1% in the leaf dry matter, which is generally reported for many deciduous tree species^2^. In our studies, however, necrotic leaf changes were observed only when the Cl^−^ concentration was not less than 2% of dry matter. Moreover, over 1% Cl^−^ concentration in the leaf dry matter was recorded in the healthy park trees. In this case, the Cl^−^ concentrations in leaves seem to be affected by aerosol deposition. Blomqvist and Johansson^4^ reported that a significant portion of the de-icing salt applied on the road might be transported by air and deposited on the ground up to 40 m from the road. High concentrations of Cl^−^ ions can be harmful to cell integrity and directly affect photosynthesis through membrane damage or enzyme inhibition when the cell vacuole can no longer sequester the incoming ions^50^. Many studies have also shown that leaf chloride concentration is most highly correlated with the degree of leaf blade damage^8,37,43^. Dmuchowski et al.^51^, assessing salt-related leaf damage in three deciduous tree species (*Tilia tomentosa* ‘Varsaviensis’, *T.* ‘Euchlora’, and *Acer saccharinum* L.), reported that trees with a leaf damage index of 4–5 contained about 40% more chloride than those with indicator values of 1–2. This is not consistent with our findings because they indicate no correlation between Cl^−^ content and degree of foliar damage. There was quite a significant variation in chloride concentration in *A. hippocastanum* leaves exhibiting the same degree of damage. Moreover, a chloride content of about 4–5% was equally often associated with 5–15% leaf blade damage, as well as 50%. Goodrich et al.^18^ also observed a large variation in the degree of aspen *Populus tremuloides* damage at the same chloride concentration since at a leaf concentration of 16 ppm Cl^−^, roadside trees exhibited a mean marginal necrosis of approximately 30% of the crown to over 90% of the damaged crown. Fostad and Pedersen^37^ reported various chloride concentrations in leaves of horse chestnut growing in Oslo centre under the influence of different sunlight intensity; however, in individual specimens, Cl^−^ ions seemed to have an even distribution in the whole crown.

Unlike chloride, sodium concentration not only increased in roadside trees but was also significantly correlated with degree of leaf damage. The intensity of these changes is illustrated by the proportion between the chloride and sodium concentrations of the tree leaves. In roadside *A. hippocastanum*, the average chloride concentration was up to 12 times higher than that of sodium ions in the leaves without damage symptoms and only 6 times in those with slight injury symptoms (up to 5% of the leaf blade), and 3–4 times in those with a greater severity of leaf tissue damage. The accumulation of Na^+^ in leaves affects photosynthetic components such as enzymes, chlorophylls and carotenoids^52,53^. Furthermore, sodium uptake is associated with changes in other cation concentrations, and the antagonistic relationships between Na^+^ and K^+^, Ca^2+^, and Mg^2+^ in several NaCl salt-stressed plants are well studied^50,54–56^. In the present study, a leaf damage of 50% and more (index 5–6) was simultaneously associated with excessive Na^+^ concentration (usually well above 1.400 mg 100 g^−1^ d.w.) and significantly reduced levels of other cations, especially potassium.

Plant physiological data indicate competition between Na^+^ and K^+^ for intracellular influx because these two cations are transported by common proteins^57^. Na^+^ also induces plasma membrane depolarisation, activating outward-rectifying K^+^ channels, which results in additional loss of cellular K^+^^58^. Thus, increasing Na^+^ content in plants normally leads to a reduction in K^+^ levels. Since K^+^ is crucial for cell osmoregulation and turgor maintenance and plays a key role in photosynthesis and protein synthesis as an activator of many cytoplasmic enzymes^59,60^, reduced K^+^ level in leaves may result in severe metabolic disorders. In addition, K^+^ leakage induced by salinity stress is often accompanied by the generation of reactive oxygen species (ROS), eventually leading to cell death^61^. Nevertheless, the dual role of ROS, as both signaling and toxic compounds, is well known^62^. Increasing evidence points to the involvement of ROS in salt stress response mechanisms and salinity tolerance^14,63^. K^+^–Na^+^ ratios have also been shown to decrease in salinity in many plant species, both herbaceous^54,64^ and woody^44,65^. Under typical physiological conditions, plants maintain a high cytosolic K^+^–Na^+^ ratio, which is necessary for optimal plant growth and leaf photosynthesis^66^. Although the analysis of plant material used in this study assessed total potassium content not only in the cytosol itself, the large disproportion of the K^+^–Na^+^ ratio between the park and roadside trees indicates significant ionic disorders caused by soil salinity. Moreover, along with the increase in necrotic symptoms observed in horse chestnuts (from marginal damage to 50% of the leaf blade), a decrease in K^+^–Na^+^ ratio was found, resulting from gradually increasing sodium content (by approx. 20% on average) and, above all, decreasing potassium content (by approx. 60%). The disruption of both cellular and whole-plant potassium homeostasis under sodium stress has been well recognised^67^. The K^+^ content declined further, altering the K^+^–Na^+^ ratio. According to Shabala et al.^68^, divalent cations such as Ca^2+^ and Mg^2+^ may prevent K^+^ leakage caused by salinity and thus support the maintenance of a high K^+^–Na^+^ ratio. However, in heavily damaged leaves (≥ 50%), along with the increase in Na^+^, there was also a decrease in Mg^2+^ and Ca^2+^ content, which might further adversely affect horse chestnut metabolism. A low Ca^2+^–Na^+^ ratio is known to increase membrane permeability, leading to an increase in passive Cl^−^ and Na^+^ transport, among others^58^. Meanwhile, at the cellular level, magnesium greatly contributes to photosynthesis and related processes in the chloroplasts, where chlorophyll-bound Mg accounts for 6–25% of the total magnesium^59^.

There are many effective ways to minimize damage caused by de-icing salts, such as watering before spring growth, chemical remediation using gypsum, and organic matter application. The use of mineral materials has also shown the positive role of gravel or coarse sand, which contribute to loosening the soil structure and thus increasing its filtration^33,69,70^.

In conclusion, NaCl used for snow removal caused a significant chloride and sodium accumulation in roadside soils and horse chestnut leaves, leading to visible damage. The accumulation of Cl^−^ and Na^+^ ions in roadside soils was accompanied by increased soil pH and reduced magnesium content. For calcium-rich soils, *A. hippocastanum* showed some tolerance to salinity since the injury symptoms occurred only at high Cl^−^ and Na^+^ ion content. Leaf damage intensity was correlated with an increase in Na^+^ content and a simultaneous decrease in K^+^ followed by Mg^2+^ and Ca^2+^. However, Na^+^ content was very high in leaves with necrotic symptoms but comparable among leaves with various intensity of leaf blade damage, i.e. 5% to more than 75%. In contrast, from necrosis covering 15% of the leaf blade, the damage severity increased with the progressive decrease in the content of K^+^ and Mg^2+^ ions. The findings suggest a need for further research to clarify the discrepancy between leaf injury intensity and chloride content. Excessive NaCl ion uptake and ionic imbalance in leaf tissues, and consequently their damage, may lead to both increased susceptibility of *A. hippocastanum* to infections and pests and weakening of tree functions in the urban environment. Overall, although some management practices, such as the addition of organic matter and supplemental watering, may help to reduce the harmful effects of soil salinity, planting horse chestnut trees in proximity to the road should be avoided.

## Supplementary Information

Supplementary Tables.
