# Supplementary material for: Effect of NaCl road salt on the ionic composition of soils and Aesculus hippocastanum L. foliage and leaf damage intensity
Source: Sci Rep. 2021 Mar 5;11:5309. doi: 10.1038/s41598-021-84541-x (PMC7935994; doi:10.1038/s41598-021-84541-x)
Supplement: Supplementary file 1 — Supplementary Tables. [file 41598_2021_84541_MOESM1_ESM.pdf]

Effect of NaCl road salt on the ionic composition of soils and *Aesculus hippocastanum* L. foliage and leaf damage intensity

Katarzyna Łuczak, Izabela Czerniawska-Kusza, Czesława Rosik-Dulewska, Grzegorz Kusza

Table 1. Granulometric composition of soil samples (Opole, Poland).

Soil samples	Depth (cm)	Percentage content of fractions with diameter (mm)				
		>2	<2	2-0.05	0.05-0.002	<0.002
1C	0-10	15	85	90	8	2
	10-30	38	62	80	10	10
2C	0-10	16	84	92	7	1
	10-30	17	83	78	17	5
3C	0-10	18	82	92	7	1
	10-30	15	85	78	18	4
4C	0-10	14	86	92	7	1
	10-30	16	84	78	16	6
5C	0-10	18	82	92	7	1
	10-30	18	82	78	16	6
6R	0-10	14	86	93	6	1
	10-30	21	79	91	8	1
7R	0-10	13	87	92	7	1
	10-30	25	75	86	11	3
8R	0-10	24	76	89	10	1
	10-30	44	56	85	11	4
9R	0-10	14	86	93	6	1
	10-30	33	67	87	10	3
10R	0-10	13	87	92	7	1
	10-30	25	75	86	11	3
11R	0-10	24	76	89	10	1
	10-30	44	56	85	11	3
12R	0-10	14	86	93	6	1
	10-30	33	67	87	10	3
13R	0-10	13	87	92	7	1
	10-30	25	75	86	11	3
14R	0-10	24	76	89	10	1
	10-30	44	56	85	11	4
15R	0-10	24	76	89	10	1
	10-30	33	67	87	10	3

Sampling sites: C – park, R - roadside.

Table 2. The pH indicator, conductivity (EC), chloride and cation concentrations in soils.

Soil samples	Depth (cm)	pH		EC ($\mu S \cdot cm^{-1}$)	Cl^-	Na^+	K^+	Mg^{2+}	Ca^{2+}
		H_2O	KCl		($mg \cdot 100g^{-1}$ d. m.)				
1C	0-10	7.49	7.09	120	0.45	4.35	3.10	2.19	1.80
	10-30	7.57	6.88	176	0.67	0.90	2.40	0.58	4.80
2C	0-10	7.62	6.69	110	0.26	1.45	5.95	1.55	1.75
	10-30	7.74	7.34	126	1.01	0.25	2.75	0.69	5.05
3C	0-10	7.66	7.03	90	0.37	2.36	4.25	1.76	1.45
	10-30	7.76	7.35	157	0.52	0.58	2.10	0.67	4.07
4C	0-10	7.59	6.94	110	0.50	2.90	4.52	1.83	1.67
	10-30	7.69	7.19	153	0.45	0.57	2.57	0.64	4.64
5C	0-10	7.62	6.89	130	0.39	1.90	5.10	1.71	1.62
	10-30	7.73	7.29	198	0.66	0.47	2.42	0.67	4.59
6R	0-10	7.80	7.40	228	1.38	12.50	3.65	0.44	3.60
	10-30	8.09	7.33	239	1.05	8.55	1.85	0.13	3.05
7R	0-10	8.04	7.31	283	1.40	26.10	2.15	0.79	3.85
	10-30	9.19	7.37	375	1.56	24.00	1.60	0.48	3.55
8R	0-10	7.86	7.27	247	1.56	5.45	8.25	0.24	5.10
	10-30	8.04	7.30	419	1.83	11.90	3.05	0.13	4.10
9R	0-10	8.02	7.28	171	2.80	17.90	4.40	0.20	2.55
	10-30	8.49	7.39	239	2.98	17.10	2.10	0.13	2.55
10R	0-10	7.92	7.35	256	1.39	19.30	2.90	0.61	3.72
	10-30	8.64	7.35	266	1.31	16.27	1.72	0.31	3.30
11R	0-10	7.95	7.29	265	1.48	15.77	5.20	0.52	4.47
	10-30	8.61	7.33	273	1.69	17.95	2.32	0.31	3.82
12R	0-10	7.94	7.27	282	2.10	11.67	6.32	0.22	3.82
	10-30	8.26	7.34	253	2.14	14.50	2.57	0.13	3.32
13R	0-10	7.97	7.32	298	1.79	18.60	3.65	0.41	3.14
	10-30	8.56	7.37	270	1.92	16.69	1.91	0.22	2.92
14R	0-10	7.93	7.32	260	2.10	17.54	4.05	0.57	4.10
	10-30	8.63	7.34	290	2.14	17.11	2.02	0.31	3.56
15R	0-10	7.94	7.28	273	1.78	13.72	5.76	0.37	4.15
	10-30	8.44	7.34	280	1.85	16.22	2.45	0.22	3.57

Sampling sites: C – park, R - roadside.
